# Supplementary material for: Metabolic Reprogramming of Glioblastoma Cells during HCMV Infection Induces Secretome-Mediated Paracrine Effects in the Microenvironment
Source: Viruses. 2022 Jan 7;14(1):103. doi: 10.3390/v14010103 (PMC8777757; doi:10.3390/v14010103)
Supplement: Supplementary file 1 [file viruses-14-00103-s001.zip › viruses-1456709-supplementary.pdf]

SUPPLEMENTARY FIGURES

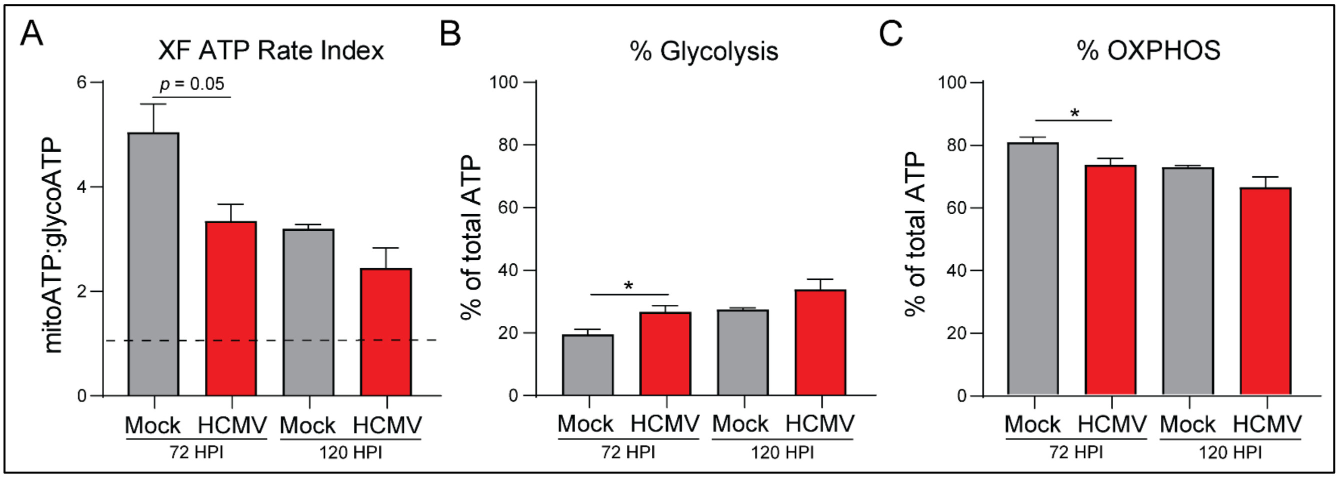

Supplementary Figure S1. HCMV infection of LN-18 cells increases glycolysis and oxidative phosphorylation ATP production. OCR and PER were measured at 72 and 120 hours post infection using the Seahorse XFe24 Real-Time ATP Rate Kit. (A) XF ATP Rate Index, (B) % Glycolysis, and (C) % Oxidative Phosphorylation were derived from the measurements illustrated in Figure 2A. All samples were normalized to cell number. Graphs represent pooled data from three independent experiments. Mean ± the SEM of at least 3 triplicates. *, $P < 0.05$.

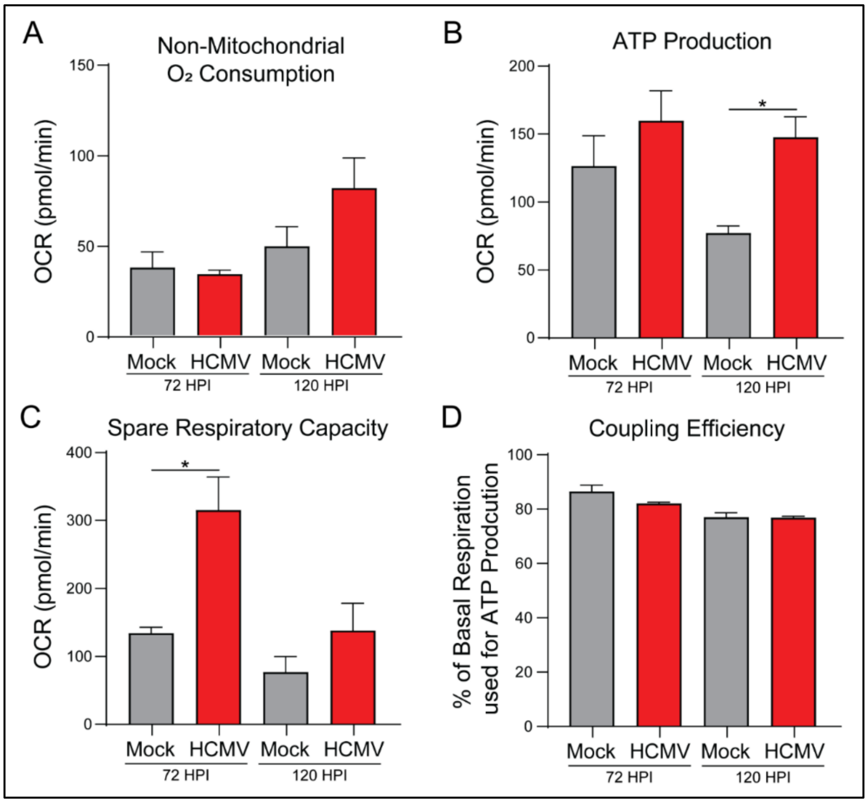

Supplementary Figure S2. HCMV infection increases oxidative phosphorylation. OCR was measured at 72 and 120 hours post infection using the Seahorse XFe24 MitoStress Kit. (A) Non-Mitochondrial Oxygen Consumption, (B) ATP Production, (C) Spare Respiratory Capacity, and (D) Coupling Efficiency were derived from these measurements. All samples were normalized to cell number. Graphs represent pooled data from three independent experiments. Mean ± the SEM of at least 3 triplicates. *, $P < 0.05$.

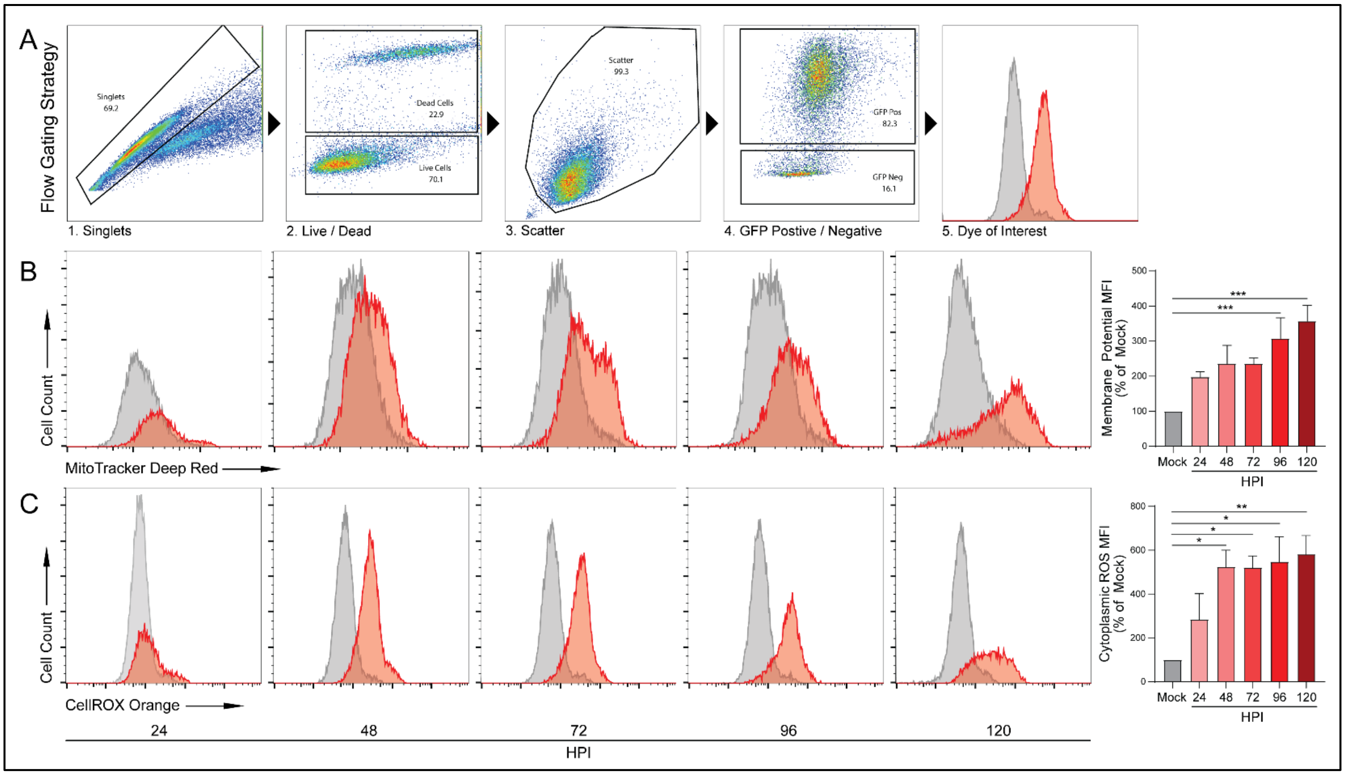

Supplementary Figure S3. Gating strategy for flow cytometric analysis of HCMV infected cells. (A) Representative flow cytometry gating strategy. (B) Mitochondrial membrane potential was quantified using MitoTracker Deep Red, a carbocyanine dye that does not rely on oxidation to become fluorescent but is dependent on mitochondrial membrane potential for its sequestration. (C) Cytoplasmic ROS was labelled with CellROX Orange and analyzed by flow cytometric analysis. Graphs represent pooled data from three independent experiments. Mean ± the SEM of at least 3 triplicates. *, $P < 0.05$; **, $P < 0.01$.

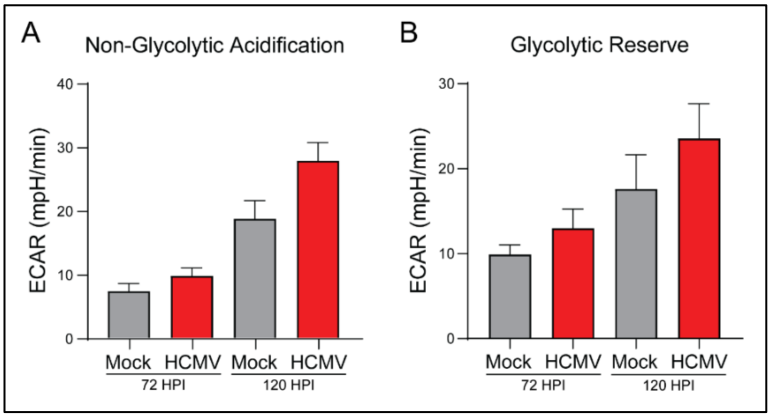

Supplementary Figure S4. The ECAR was measured at 72 and 120 hours post infection using the Seahorse XFe24 Glycolysis Stress Kit. (A) Non-glycolytic acidification and (B) Glycolytic Reserve were derived from these measurements. All samples were normalized to cell number. Graphs represent pooled data from three independent experiments. Mean ± the SEM of at least 3 triplicates.

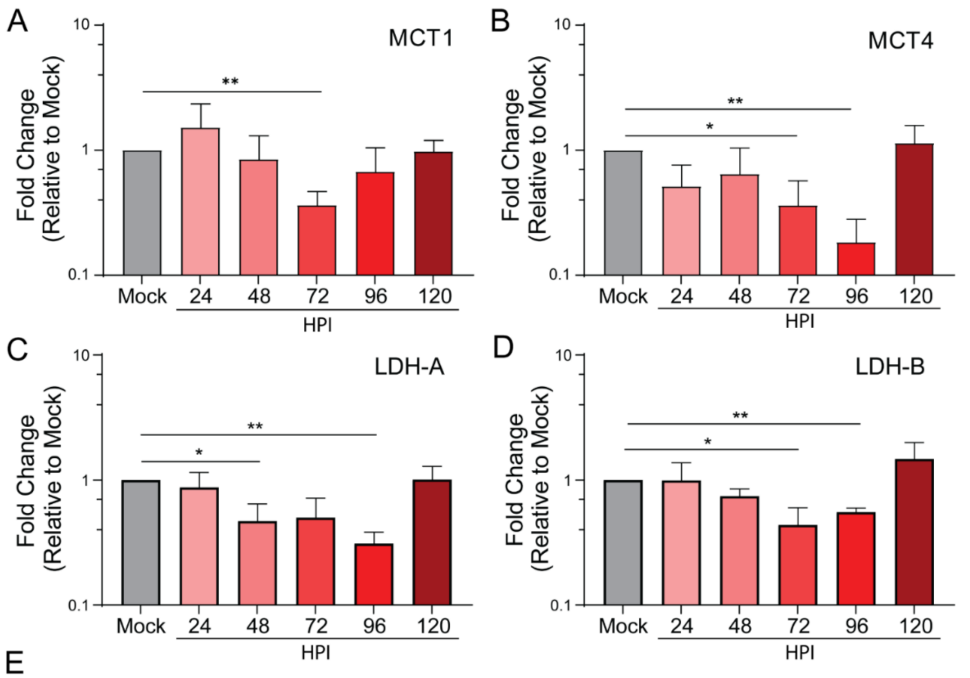

E

RNA-Seq Differential Expression: 72 Hour G44 HCMV-Infected vs Mock-Infected

Gene ID	Fold Change	Adjusted P Value	Significant
SLC16A1 (MCT1)	0.52	0.00000	Yes
SLC16A3 (MCT4)	1.77	0.09689	No
LDH-A	0.45	0.00000	Yes
LDH-B	0.77	0.01903	Yes

Supplementary Figure S5. HCMV infection downregulates expression of key lactate regulatory genes. LN-18 cells were mock infected or HCMV infected and RNA was collected at the indicated timepoints following infection. RT-qPCR was conducted and fold changes relative to mock infected cells were calculated using the delta-delta Ct method. Following infection, cells displayed decreased gene expression for two key lactate transporters (A) MCT1 and (B) MCT4; and the two lactate dehydrogenase subunits (C) LDH-A (D) LDH-B. (E) RNA-sequencing was conducted on G44 cells either mock-infected or 72 hours post HCMV infection. Graphs represent pooled data from three independent experiments. Mean ± the SEM of at least 3 triplicates. *, $P < 0.05$; **, $P < 0.01$.
